# Substitution matrix based color schemes for sequence alignment visualization

**DOI:** 10.1186/s12859-020-3526-6

**Published:** 2020-05-24

**Authors:** Patrick Kunzmann, Benjamin E. Mayer, Kay Hamacher

**Affiliations:** Department of Computational Biology and Simulation, TU Darmstadt, Schnittspahnstraße 2, Darmstadt, 64287 Germany

**Keywords:** Open source, Python, Color space, Optimization, Sequence alignment

## Abstract

**Background:**

Visualization of multiple sequence alignments often includes colored symbols, usually characters encoding amino acids, according to some (physical) properties, such as hydrophobicity or charge. Typically, color schemes are created manually, so that equal or similar colors are assigned to amino acids that share similar properties. However, this assessment is subjective and may not represent the similarity of symbols very well.

**Results:**

In this article we propose a different approach for color scheme creation: We leverage the similarity information of a substitution matrix to derive an appropriate color scheme. Similar colors are assigned to high scoring pairs of symbols, distant colors are assigned to low scoring pairs. In order to find these optimal points in color space a simulated annealing algorithm is employed.

**Conclusions:**

Using the substitution matrix as basis for a color scheme is consistent with the alignment, which itself is based on the very substitution matrix. This approach allows fully automatic generation of new color schemes, even for special purposes which have not been covered, yet, including schemes for structural alphabets or schemes that are adapted for people with color vision deficiency.

## Background

**Fig. 1:**
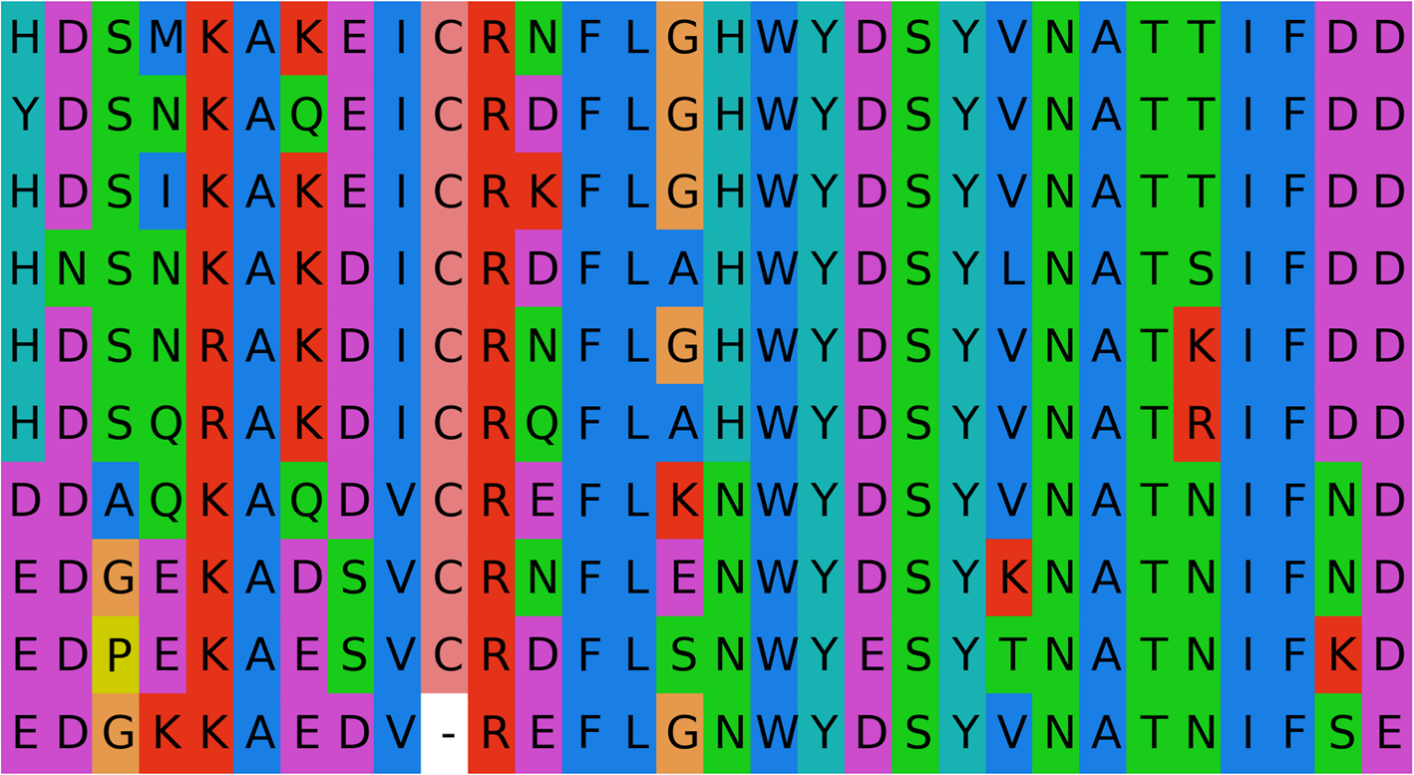
Visualization of a multiple sequence alignment. The alignment uses the default ClustalX color scheme [3]. The alignment is an excerpt of a multiple protein sequence alignment of bacterial luciferases (*luxA* gene product). The figure was created with Biotite [13]

Typically, visualization of multiple protein sequence alignments colors the amino acid symbols according to some kind of (chemical) property. Examples for software using this visualization technique are *MSAViewer* [1], *JalView* [2] or *ClustalX* [3]. Figure 1 shows an alignment using the default *ClustalX* color scheme depicting the chemical characteristics of the amino acids. Typically, such a color scheme is created manually by a professional using their intuition and knowledge about any characteristics to be emphasized.

However, the intuition might not reflect the biological similarity of two amino acids in terms of evolutionary distance, as defined by amino acid substitution scores (and thus probabilities).

If the color in an alignment visualization is used to depict general physical amino acid characteristics, in contrast to a specific measurable or computable quantity, e.g. hydropathy or secondary structure propensity, we reckon substitution matrices would be a more meaningful and reproducible basis for color scheme creation.

In this article we describe a method and corresponding software that is able to directly generate color schemes from a substitution matrix in an automatic manner: The aim is to find those colors for each symbol, for which the pairwise perceptual color differences correspond to pairwise symbol distances. The more similar two symbols are, the more similar the assigned colors should be. We formulated this criterion as score function and used a *simulated annealing* [4] optimizer, that searches for the optimal colors for which the score function is minimal.

### Color space

In order to find the optimal colors that minimize the score function, a *color space* to search in is required. A color space is a 3-dimensional vector space where a point represents a corresponding color. The arguably most common color space is *RGB* or, more exactly, *standard RGB* (*sRGB*) [5]. It defines a color as an addition of red, green and blue light. Although this color space is very useful in the context of display devices, it lacks perceptual uniformity [6]: changes of an RGB color value do not result in a proportional perceptual change.

In contrast, the CIE L*a*b* color space [7] shows perceptually approximately uniform characteristics, i.e. the perceptual difference of two colors is approximately proportional to the Euclidean distance of the color components. The L*a*b* color space consists of these three components:

- L* - The lightness of the color. 0 is completely black and 100 is completely white.
- a* - The green-red component. Green is in the negative direction, red is in the positive direction.
- b* - The blue-yellow component. Blue is in the negative direction, yellow is in the positive direction.

**Fig. 2:**
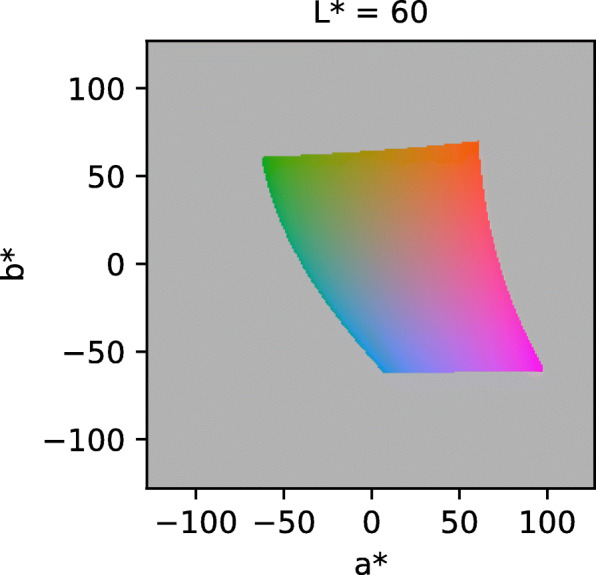
*RGB* subspace of the L*a*b* color space. The plot shows a L*a*b* space cross section at a fixed L* value. The color depicts the *RGB* value at the respective position. The gray area cannot be converted into *RGB*

While a* and b* values are theoretically unlimited in either direction, only a limited L*a*b* subspace is displayable by devices and thus can be converted into *sRGB* (Fig. 2).

## Implementation

### Score function

Our method should find similar colors for similar symbols and distant colors for dissimilar symbols. In order to quantify this objective, differences are computed via a score function. The score function takes a combination of CIE L*a*b* colors (*color conformation*), represented as a (*N*×3)-matrix, and returns a scalar score value. *N* is the number of symbols in the alphabet (20 for amino acids).

In the following <*X*> denotes the arithmetic mean of all elements of a matrix: $\left < X \right > := \frac {1}{n} \sum _{ij} X_{ij}$. For a triangular matrix only the non-zero diagonals are taken into account; *n* is the number of respective matrix elements.

#### Construction of distance matrices

First, the input substitution matrix *M* is converted into a distance matrix *D*^′^. *D*^′^ is triangular to remove redundant entries, since the distance is commutative.

$$ D'_{ij} = \left\{\begin{array}{ll} \left((M_{ii} - M_{ij}) + (M_{jj} - M_{ji}) \right) / 2, & \text{for} \; j \leq i \\ 0, & \text{for} \; j > i \end{array}\right. $$

For any substitution matrix *M*, *M*_*ii*_ should be the maximum value in the row/column *i*, otherwise a symbol would be more similar to another symbol than to itself. Consequently, the main diagonal of *D*^′^ contains only zeros.

To be agnostic about the magnitude of substitution scores in *M*, *D*^′^ is scaled, so that the average distance is 1.

$$D = \frac {D'} {\left< D' \right>} $$

#### Calculation of the perceptual difference matrix

On the other hand, the triangular matrix *C* denotes the pairwise perceptual differences of the L*a*b* colors for each pair of symbols. Our implementation uses the *CIEDE2000* formula [8], which is omitted for brevity. However, the formula can be approximated as the Euclidean distance [7]:

$$ C_{ij} \approx \sqrt{(L^{*}_{i} - L^{*}_{j})^{2} + (a^{*}_{i} - a^{*}_{j})^{2} + (b^{*}_{i} - b^{*}_{j})^{2}} $$

Note, while *D* is constant, *C* is dependent on the (current) color conformation.

In order to relate the L*a*b* color differences in *C* to the distances in *D*, a scale factor *f*_*s*_ is introduced. *f*_*s*_ is the proportion of the average distance in *D* to the average difference in *C*:

$$ f_{s} = \frac{\left< D \right>}{\left< C \right>} = \frac{ 1} { \frac{1}{n} \sum_{ij} C } = \frac{ n} { \sum_{ij} C_{ij} } $$

As *C* is variable, *f*_*s*_ also dynamically changes during our optimization runs.

#### Score function

The score function *S*_*T*_ is a sum of two terms: a sum of harmonic potentials between each pair of symbols *S*_*H*_ and a linear *contrast scoreS*_*C*_:

$$S_{T} = S_{H} + S_{C} $$

The harmonic potentials are used to adjust the relative color differences in accordance with the substitution matrix. The equilibrium distance of each potential corresponds to the distance in the distance matrix *D*:

$$S_{H} = \sum_{ij} \left(f_{s} C_{ij} - D_{ij} \right)^{2} $$

However, this term is not sufficient to create an appealing color scheme: due to the scale factor *f*_*s*_, a scheme with a small average color difference would get the same score as a scheme with a high average color difference. In consequence low contrast color schemes could arise. In order to favor high contrast color schemes the contrast score *S*_*C*_ is introduced. A reciprocal function based on the average color difference is used here. The *contrast factorf*_*c*_ is a user-supplied parameter for weighting this term:

$$S_{C} = \frac{f_{c}}{\left< C \right>} $$

### Optimization

The question, which color conformation minimizes the score function, is a (*N*×3)-dimensional, continuous optimization problem. As the optimization landscape can be restricted via user input, we face the problem of a non trivially bounded and, depending on the constraint, possibly non-convex optimization problem. In general, obtaining an exact solution in a non-convex, continuous problem setting is computationally hard, as shown for the example of pair potentials in atomic clusters [9]. Therefore, we resort to heuristic optimization, namely *simulated annealing* (SA) [4], as described in Algorithm 1. 
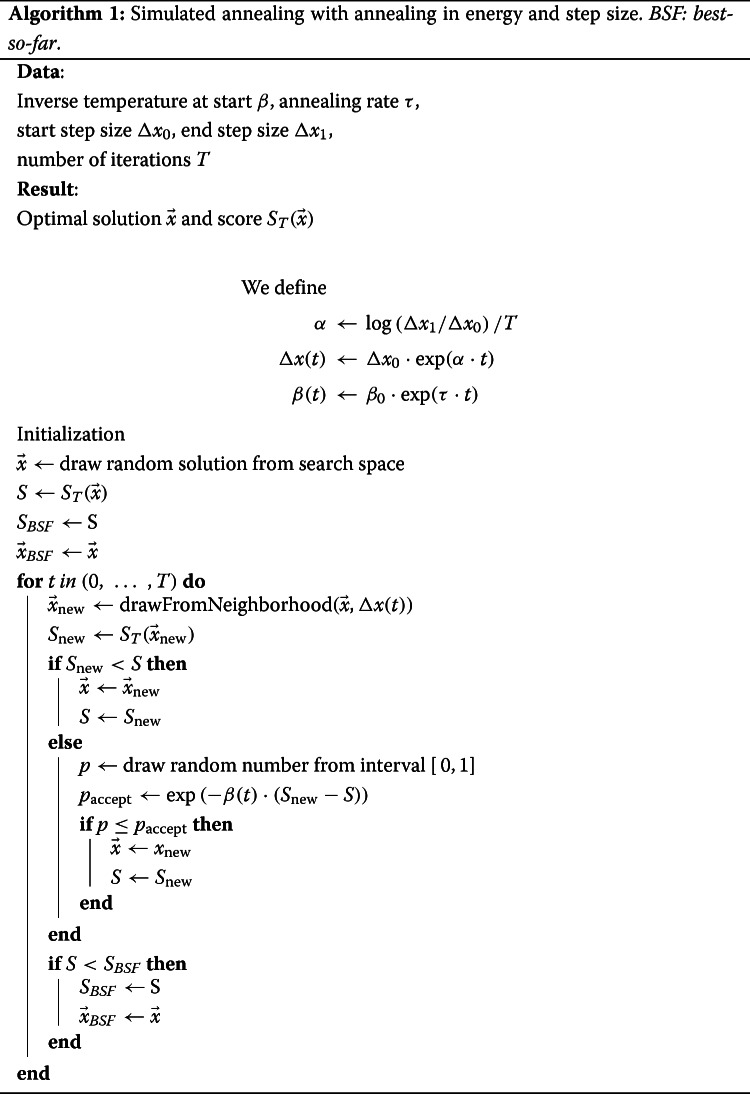


The SA algorithm samples the search space, which is a vector space consisting of color space vectors $ \vec {x} = \left (L^{*}_{1}, a^{*}_{1}, b^{*}_{1}, \hdots, L^{*}_{N}, a^{*}_{N}, b^{*}_{N}\right) $.

Sampling is realized based on a neighborhood definition in color space. For the space of color space vectors this neighborhood is defined by all valid colors reachable by adding perturbations of the color values drawn from a uniform distribution $U(-\Delta \vec {x}(t), \Delta \vec {x}(t))$, excluding the user specified region.

Initially the search space is sampled with a high temperature, so the algorithm has the ability to escape local minima or even jump over large sections of the search space. By gradually limiting this behavior, which is specified by the annealing schedule *β*(*t*) (quantified by *τ*), the algorithm converges to a suitable optimal solution – guarantees about the found optima’s quality, however, cannot be given due to the heuristic approach of SA. Yet, the convergence towards the global optimum in infinite time is a proven quality of this algorithm [10, 11]. Therefore, after a sufficiently long run time a non-optimal, but nonetheless *good* solution is found.

Typically SA is used for combinatorial optimization problems, i.e. problems defined on a discrete search space, e.g., the traveling salesman problem [4]. Since its inception SA has also been adapted to various continuous optimization problems.

As it turns out a robust adaption of SA for the continuous problem discussed here, is realized by simply doing an annealing in both the temperature and step size.

We run an ensemble of SA instances, meaning multiple independent instances with different random number generator seeds. The best found solution and score seen during a single SA run are captured in the variables $\vec {x}_{BSF} $ and *S*_*BSF*_.

These quantities are referred to as the *best-so-far* solution and score. After the last iteration the optimal solution is given as the minimum of the *best-so-far* solutions within the ensemble of optimizers. In our implementation we also store the ensemble maximum which is given by the maximum of the worst seen solutions over the algorithm run, which we neglected to include in Algorithm 1 in favor of simplicity, as in principle a broad variety of run features could be stored in the same way as the *best-so-far* solution. Furthermore an ensemble average and standard deviation are stored. These two properties are used for further quality analysis of the SA run.

### Software

We have implemented the method for color scheme generation in the Python package Gecos. In addition to the more flexible Python API, the package offers a command line interface (CLI) for simple color scheme generation. Either way, the alphabet, substitution matrix and color subspace can be customized for the purpose of the user. By default the software creates a color scheme for the *BLOSUM62* matrix [12]. The CLI saves the generated color scheme in JSON format, containing the RGB color code for each symbol. The JSON format can be directly used for alignment visualization in Biotite [13]. For usage in other visualization software the color codes must be inserted into the input format of the respective program.

## Results and discussion

### Optimization

**Fig. 3:**
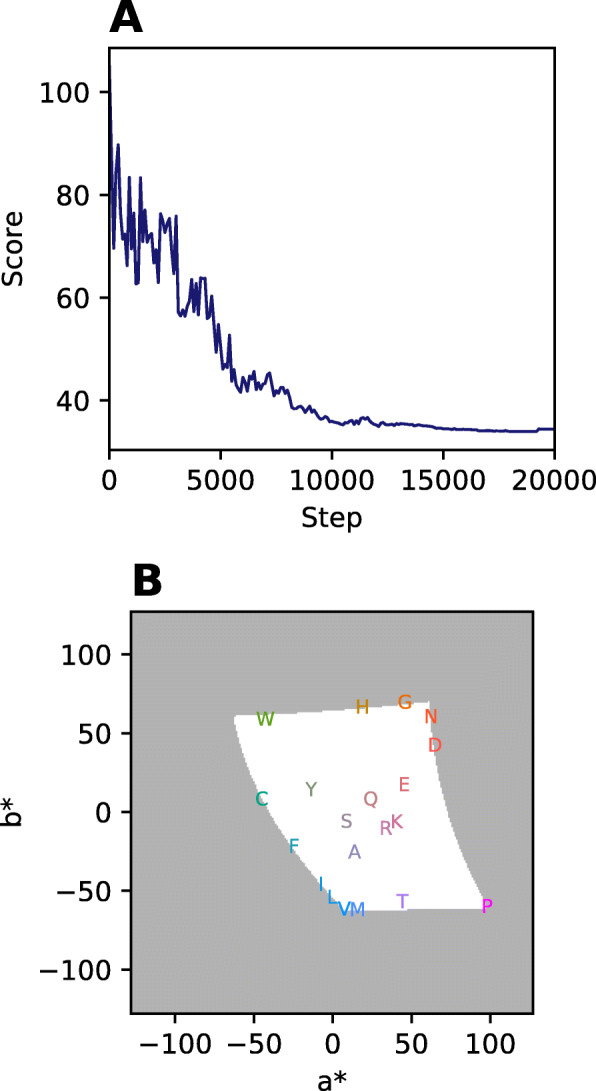
Color scheme optimization. For the sake of clearness the color space was constrained to a single lightness value of L* = 60. Otherwise the shown results use the default parameters of the CLI invocation. **a** The score trajectory is plotted against the number of optimization steps. For clarity only every 100th step is displayed. **b** The plot shows the final color conformation after optimization. The white area displays the allowed color space. The position of a symbol depicts its a* and b* value

Figure 3 shows the improvement of the score during the optimization process and the resulting color conformation. Generally, the evaluation whether the score of the final color conformation is near the global optimum is not directly possible, since the calculation of the score at the global optimum requires knowledge of the optimal color conformation.

**Fig. 4:**
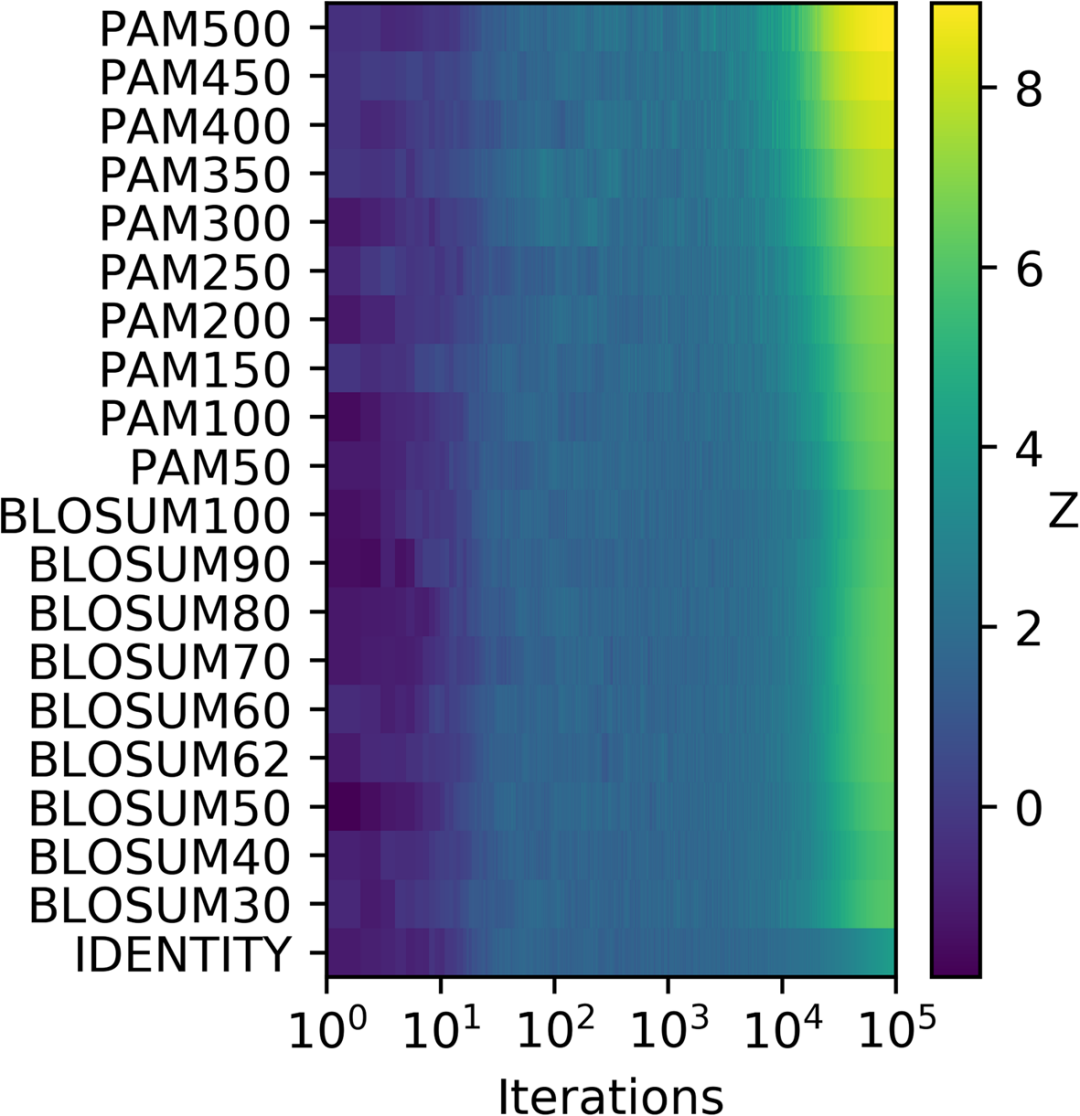
Z-scores *Z* for different substitution matrices and rising iterations. To analyze the quality of the implemented SA algorithm, the optimization has been applied to a selection of substitution matrices. A Z-score like metric is used here to visualize the reached score relative to randomized sampling of the search space. The iterations are specified on the x-axis on a logarithmic scale, whereas the matrices are given on the y-axis

In our approach we compared the outcome of a reference optimization run with random sampling in the color conformation space. To show the robustness of the implemented optimization technique with regards to user input we benchmarked on a selection of substitution matrices. This is visualized in Fig. 4, where a Z-score-like empirical measure

$$ Z(t) := - \frac{ \langle S_{T}\rangle_{SA}(t) - \mu(t) }{ \sigma(t) } $$

of the optimizer quality relative to the random sampling is plotted over increasing iterations. At a given iteration the difference between the reached average scores of the optimizer and random sampling is normalized to the standard deviation *σ*(*t*) seen by random sampling so far. The negative sign of *Z*(*t*) is explained by the fact that our score *S*_*T*_ gets smaller for a better solution, so the scores reached by the optimizer should be smaller then the ones reached by sampling, in which case *Z*(*t*) is positive.

While for the optimizer the averaging is done over the ensemble of optimizers, the mean *μ*(*t*) and standard deviation *σ*(*t*) for the random sampling is calculated using the so far sampled data. Here the rationale is that a randomized sampling in color space should eventually stabilize, by thus producing a distribution of solutions that can be used as ground truth to separate from steered processes. The SA algorithm is exactly such a steered process, as it essentially does random sampling, yet from a local neighborhood instead of the whole search space, and is biased towards lower scores.

If the optimizer was an unsteered process one would expect low values for *Z* as this would coincide with the optimizer not finding better values then a randomized sampling. This behavior is seen in Fig. 4 for the early iterations.

However, with increasing iterations *Z* is getting successively higher until finally reaching a region of *Z*≈6 meaning that on average SA reaches a score that is six standard deviations *better* then purely randomized sampling. While it seems that for some of the *PAM* substitution matrices the algorithm performs better then for the others, the fact stands that overall a superior solution is reached, independently of the user selected input. The only matrix for which we see significantly different behavior in Fig. 4 is the *IDENTITY* matrix. This is expected, since the optimal distance between symbols is equidistant for all pairs of symbols here. Therefore, finding an optimal solution should be more difficult, which accounts for the overall lower Z-score. Yet, even here the SA optimizer outperforms the random sampling and eventually reaches a region with *Z*≈4.

### Color schemes

**Fig. 5:**
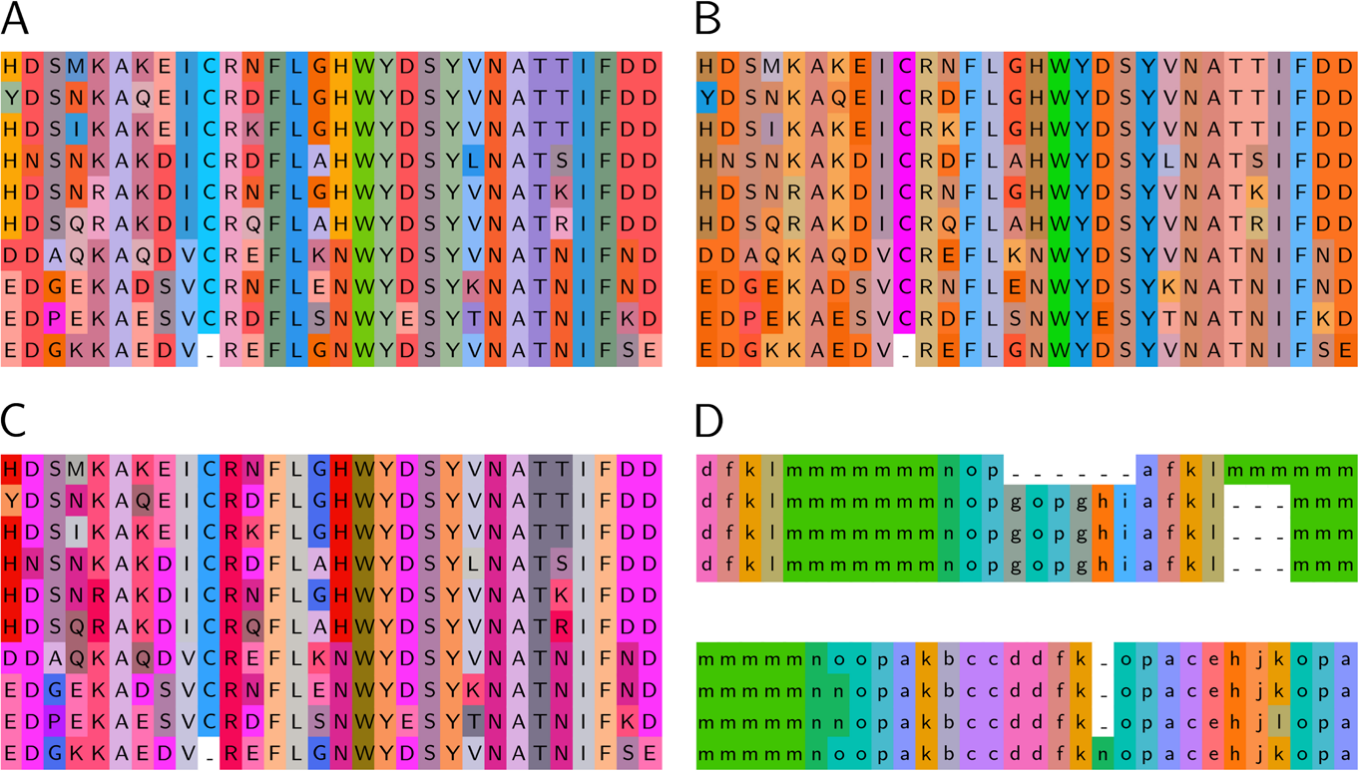
Alignments with generated color schemes. Gaps have no color assigned, since a gap means the absence of a symbol. **a** The alignment from Fig. 1 is visualized with a color scheme generated with Gecos. The L* value was constrained between 60 and 75. Otherwise the shown results use the default parameters of the CLI invocation. **b** Generated color scheme for *PAM250* instead of *BLOSUM62*. **c** Red-green color vision deficiency adapted color scheme by removing the green part of the color space. The range for the L* value was broadened from 50 to 80. **d** Color scheme for the *protein blocks* (PB) structural alphabet. The PB sequences were calculated from structures of lysozyme variants. The substitution matrix and references angles were taken from Barnoud et al. [19]

Figure 5a shows a color scheme that was created via the Gecos CLI for the *BLOSUM62* substitution matrix. The lightness range was constrained to 60<*L*^∗^<75, but otherwise the default CLI parameters were used. The constraint is necessary as the scheme would not look appealing if the whole lightness range - from black to white - would have been included. On the other hand we found that fixing the lightness to a single value results in schemes that have a low contrast. Thus, we recommend using a lightness interval between 15 and 30.

#### Dependence on substitution matrix

The generated color scheme is strongly dependent on the used substitution matrix. This becomes more clear when comparing the *BLOSUM62* based color scheme (Fig. 5a) with a *PAM250* [14] based scheme (Fig. 5b). Compared to *BLOSUM62*, *PAM250* assigns an extraordinarily low score for substitution of tryptophane and cysteine with any other amino acid. Consequently, the *PAM250* based color scheme especially highlights tryptophane and cysteine, while the contrast between the other symbols is relatively low.

#### Constraints in a*b* dimensions

In addition to constraining the lightness L*, it can also be reasonable to limit the color space in the a* and b* dimensions. The use cases include

- limiting the saturation of the colors,
- creating a scheme with a specified hue,
- taking color vision deficiency into account.

The most common color vision deficiency is the red-green color blindness, that affects approximately 8 % of the male and approximately 0.5 % of the female population [15–17]. In order to create a color scheme, that is more friendly to red-green color vision deficient people, we ran an optimization on a color subspace where the green part is omitted, i.e. *a*^∗^>0 (Fig. 5c). As the reduced size of the color space would inherently cause a loss of contrast, the available lightness interval is increased to 50<*L*^∗^<80.

#### Color schemes for exotic alphabets

Since the described method does not require professional knowledge about the properties of each symbol, but merely needs a substitution matrix, it can be easily transferred to alphabets other than amino acids.

Structural alphabets are such a use case. Structural alphabets encode local protein structure properties, e.g. backbone dihedral angles or pseudo bond angles, into symbols that represent the local conformation. In this way a 3D structure can be converted into a symbol sequence. Creating a color scheme for a structural alphabet can be difficult, because the symbols do not exhibit such tangible properties like charge or hydrophobicity. However, if a substitution matrix is available for the structural alphabet, our approach is able to automatically compile a color scheme.

The *protein block* (PB) alphabet is such a structural alphabet [18]. It contains 16 symbols from a to p, each representing a different local peptide backbone conformation. Figure 5d shows a scheme based on the PB substitution matrix [19], generated with Gecos.

### Comparison with existing amino acid color schemes

**Fig. 6:**
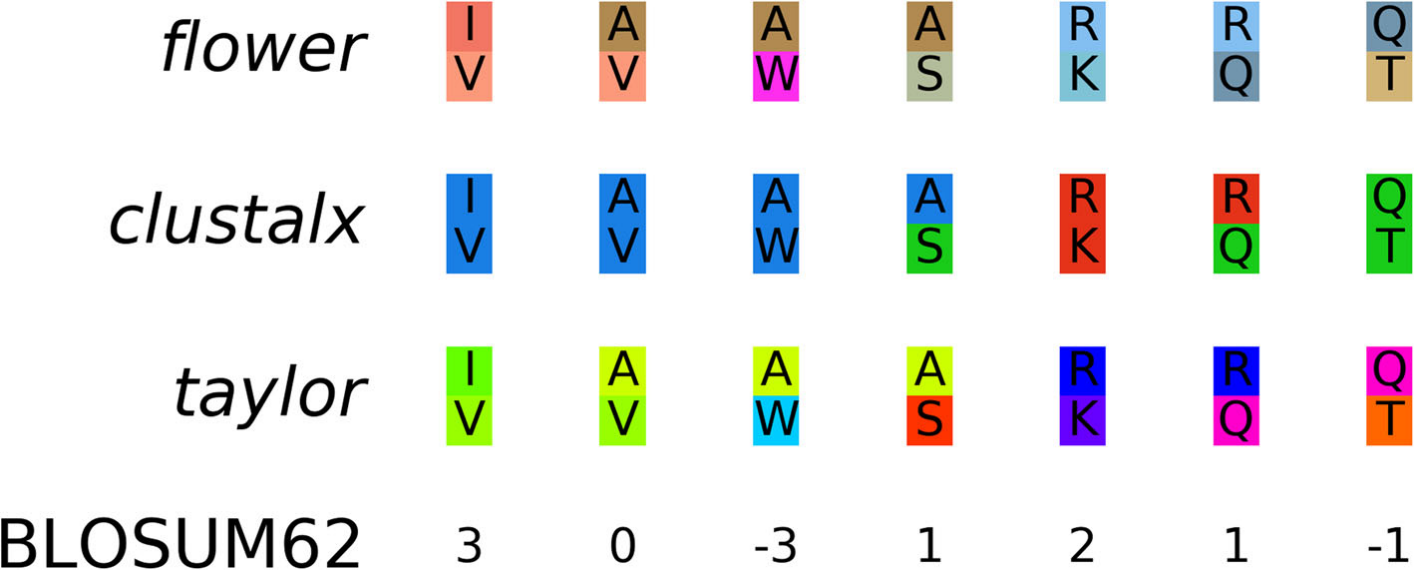
Color scheme comparison. For chosen amino acid pairs, the plot shows their colors in different schemes and the *BLOSUM62* score [12] of the respective pair

Our method aims to generate color schemes that depict evolutionary similarity of amino acids better than the existing, manually created ones. Based on chosen amino acid pairs, we compared a color scheme generated by *Gecos*, namely *flower* implemented in *Biotite* [13], with two traditional color schemes: the default one from *ClustalX* [3] (denoted as *clustalx*) and the *Taylor* [20] color scheme (denoted as *taylor*) (Fig. 6).

Both *clustalx* and *taylor* use identical or similar colors, respectively, for alanine (A) valine (V) and isoleucine (I). However, valine is much more (evolutionary) similar to isoleucine than to alanine, according to the BLOSUM62 [12] matrix. In fact, alanine is slightly more similar to serine (S) than to valine. Still, *clustalx* and *taylor* assign very dissimilar colors to alanine and serine, as alanine is classified as hydrophobic in contrast to the more polar serine. *clustalx* also uses the same color for alanine and tryptophan (W), because both amino acids are hydrophobic. However, the similarity score between both residues is exceptionally low.

A similar result can be seen for the positively charged and polar residues: although all three color schemes are able to depict the high similarity between lysine (K) and arginine (R), as both are usually positively charged, *clustalx* and *taylor* use highly different colors for the arginine-glutamine (Q) pair, as glutamine is neutral. Instead, they use identical or similar colors, respectively, for the glutamine-threonine (T) pair. Although both are uncharged polar residues, glutamine is closer to arginine than to threonine from an evolutionary point of view.

In contrast, *flower* does not categorize amino acids into groups based on e.g. hydrophobicity or residue size, but only uses colors based on pairwise amino acid similarities. Consequently, falsely suggested similarities, e.g. the alanine-tryptophan pair in *clustalx*, and falsely suggested dissimilarities, e.g. the alanine-serine pair in *clustalx* and *taylor*, are less likely to occur. Hence, *flower* is more reliable for visual analysis of multiple sequence alignments, when it comes to identification of evolutionary distant or close regions.

## Conclusion

Our method enables the user to create a new color scheme for sequence alignments in a fast and easy way - may it be for special purposes like exotic alphabets, where no color scheme does exist yet, or simply because a new scheme appeals more than the existing ones.

Traditional color schemes depict specific characteristics of amino acids, for example hydrophobicity, charge, secondary structure propensities or a combination of these. Taking a substitution matrix as basis for the color scheme incorporates more evolutionary meaning. Although a specific property cannot directly be read from the color, this novel approach depicts the similarity of amino acids better in terms of substitution probability. Therefore, this method is consistent with the alignment, which itself is based on substitution probabilities and thus on molecular evolution.

## Availability and requirements

**Project name:** Gecos **Project home page:**https://gecos.biotite-python.org/**Operating system(s):** Windows, OS X, Linux **Programming language:** Python **Other requirements:** At least Python 3.6, the packages *biotite*, *numpy* and *scikit-image* must be installed **License:** BSD 3-Clause **Any restrictions to use by non-academics:** None

## Data Availability

The Gecos source code is hosted at https://github.com/biotite-dev/gecos and its official documentation at https://gecos.biotite-python.org/. The version 1.1, that was used in this study, is available as archive [21].

## Abbreviations

API: Application programming interface
CIE: International commission on illumination
CLI: Command line interface
SA: Simulated annealing
sRGB: Standard RGB

## References

[1] Yachdav G, Wilzbach S, Rauscher B, Sheridan R, Sillitoe I, Procter J, Lewis SE, Rost B, Goldberg T (2016). MSAViewer: Interactive JavaScript visualization of multiple sequence alignments. Bioinformatics.

[2] Waterhouse AM, Procter JB, Martin DMA, Clamp M, Barton GJ (2009). Jalview Version 2-A multiple sequence alignment editor and analysis workbench. Bioinformatics.

[3] Larkin MA, Blackshields G, Brown NP, Chenna R, Mcgettigan PA, McWilliam H, Valentin F, Wallace IM, Wilm A, Lopez R, Thompson JD, Gibson TJ, Higgins DG (2007). Clustal W and Clustal X version 2.0. Bioinformatics.

[4] Kirkpatrick S, Gelatt CD, Vecchi MP (1983). Optimization by simulated annealing. Science.

[5] IEC: IEC 61966-2-1. Multimedia systems and equipment - Colour measurement and management - Part 2-1: Colour management - Default RGB colour space - sRGB. 1999. https://webstore.iec.ch/publication/6169.

[6] Bernard J, Steiger M, Mittelstädt S, Thum S, Keim D, Kohlhammer J. A survey and task-based quality assessment of static 2D colormaps. In: Visualization and Data Analysis 2015, vol. 9397: 2015. p. 247–62. 10.1117/12.2079841.

[7] CIE: ISO/CIE 11664-4. Colorimetry – Part 4: CIE 1976 L*a*b* colour space. 2019. https://www.iso.org/standard/74166.html.

[8] CIE: CIE 142-2001. Improvement to industrial colour-difference evaluation. 2001. http://cie.co.at/publications/improvement-industrial-colour-difference-evaluation.

[9] Wille LT, Vennik J (1985). Computational complexity of the ground-state determination of atomic clusters. J Phys A Math Gen.

[10] Aarts E, Lenstra JK (1997). Local Search in Combinatorial Optimization, 1st edn..

[11] Laarhooven PJM, Aarts EHL (1987). No Title.

[12] Henikoff S, Henikoff JG (1992). Amino acid substitution matrices from protein blocks,. Proc Natl Acad Sci U S A.

[13] Kunzmann P, Hamacher K. Biotite: A unifying open source computational biology framework in Python. BMC Bioinformatics. 2018; 19(1). 10.1186/s12859-018-2367-z. 10.1186/s12859-018-2367-z PMC6167853 30285630

[14] Dayhoff MO. A Model of Evolutionary Change. In: Proteins in Atlas of Protein Sequence and Structure, vol. 5 Suppleme: 1978. p. 345–52.

[15] NEI. Facts about color blindness. https://nei.nih.gov/health/color_blindness/facts_about. Accessed 28 June 2019.

[16] Kovalev VA. Towards image retrieval for eight percent of color-blind men. In: Proceedings - International Conference on Pattern Recognition, vol. 2: 2004. 10.1109/ICPR.2004.1334414.

[17] Al-Aqtum Musa T., Al-Qawasmeh Mohammed H. (2000). Prevalence of Colour Blindness in Young Jordanians. Ophthalmologica.

[18] De Brevern AG, Etchebest C, Hazout S. Bayesian probabilistic approach for predicting backbone structures in terms of protein blocks. Protein Struct Funct Genet. 2000; 41(3):271–87. https://doi.org/10.1002/1097-0134(20001115)41:3〈271::AID-PROT10〉3.0.CO;2-Z. 10.1002/1097-0134(20001115)41:3<271::aid-prot10>3.0.co;2-z 11025540

[19] Barnoud J, Santuz H, Craveur P, Joseph AP, Jallu V, de Brevern AG, Poulain P (2017). PBxplore: a tool to analyze local protein structure and deformability with Protein Blocks. PeerJ.

[20] Taylor WR (1997). Residual colours: A proposal for aminochromography. Protein Eng.

[21] Kunzmann P, Mayer B. Gecos 1.1.0 repository snapshot. 2019. 10.5281/zenodo.3490531.

